# A Visual Question Answering Dataset for Benchmarking Vision-Language Models in Plant Science

**DOI:** 10.1038/s41597-026-07779-y

**Published:** 2026-08-10

**Authors:** Syed Nazmus Sakib, Nafiul Haque, Mohammad Zabed Hossain, Shifat E. Arman

**Affiliations:** 1 Department of Robotics and Mechatronics Engineering, University of Dhaka, Dhaka, Bangladesh; 2 Department of Botany, University of Dhaka, Dhaka, Bangladesh

**Keywords:** Biotic, Computer science

## Abstract

Existing plant-disease datasets target classification and detection, leaving vision-language models unable to support interactive, reasoning-based diagnosis. To address this, we present PlantExpertVQA, a large-scale visual question answering (VQA) dataset designed to advance vision-language models for agricultural decision-making. It is compiled from 45 open-source datasets, including the widely used PlantVillage corpus, and comprises 765,186 high-quality question-answer (QA) pairs grounded over 150,841 images spanning 38 crop species and 89 disease conditions. Questions are organized into 3 levels of cognitive complexity and 9 distinct categories. Each was phrased following expert guidance and generated via an automated two-stage pipeline: template-based QA synthesis from image metadata, followed by multi-stage linguistic re-engineering. The dataset was iteratively reviewed by domain experts for scientific accuracy and relevance. We find that current frontier vision-language models, including recent open-source instruction-tuned multimodal LLMs, perform poorly on PlantExpertVQA. However, parameter-efficient fine-tuning of a compact 2B-parameter model on a small fraction of the dataset yields substantial improvements across all question categories, demonstrating its effectiveness for domain adaptation.

## Background & Summary

Plant diseases threaten global food security and farm productivity. Research indicates that plant pests and diseases are responsible for the loss of as much as 30% of global food crop yields annually^1^. This results in famine, malnutrition, and food insecurity for hundreds of millions of people worldwide. In most cases pest and fungal invasions spread rapidly due to diagnostic delay. Therefore, precision tools are now needed for early symptoms detection and targeted interventions.

Machine learning has advanced plant pathology by enabling automated identification of disease symptoms. Convolutional neural networks and transformer models can reliably detect leaf diseases in various crop species^2–5^. However, most existing frameworks focus only on classification and do not provide insights into symptom causation or context.

Visual Question Answering (VQA)^6,7^ combines image understanding with natural language processing to answer queries about visual content. VQA databases go beyond classification by allowing interactive question-answering^8^. This allows trained models to capture complex relationships in the images. As such, the application of VQA now spans multiple domains. These include: educational tools^9,10^, customer service systems^11,12^, and autonomous driving^13^ etc. In particular, VQA shows exceptional potential in the field of pathological diagnosis and health inquiry^14,15^. Current medical VQA benchmarks include PMC-VQA^14^, SLAKE^16^, Path-VQA^17^, and VQA-RAD^18^. However, these datasets are focused on medical diagnostics.

In agriculture, existing popular datasets like PlantVillage^4^, PlantDoc^19^, and PlantSeg^20^ focus on classification or segmentation tasks. While they support disease detection, they do not enable interactive reasoning through question-answer formats. Recent systems like AgroGPT^21^, LLaVa-PlantDiag^22^ incorporate VQA models with the PlantVillage dataset. But these resources use generalized large language models for text generation and lack rigorous expert verification.

**Fig. 1:**
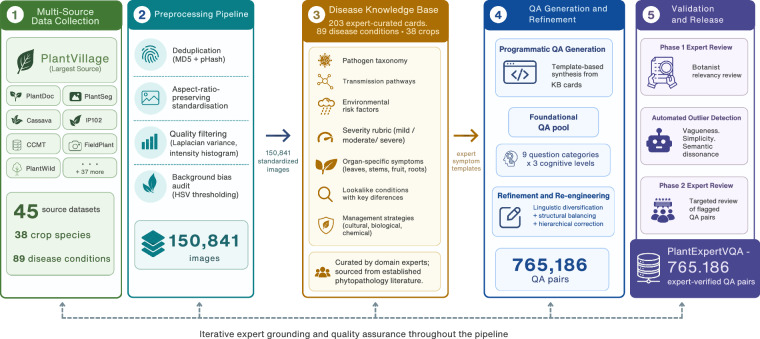
Overall Methodology of PlantExpertVQA creation.

To address these gaps, we introduce PlantExpertVQA, a domain-specific visual question answering dataset for plant disease diagnosis. The dataset is built on a compilation of 45 open-sourced datasets featuring explicit, well-curated hierarchical annotations that underwent extensive preprocessing and standardization. It contains 765,186 question-answer pairs grounded over 150,841 images across 38 unique crop species and 89 disease conditions, covering nine distinct question categories under three levels of cognitive complexity. Each question was naturally phrased and tailored through expert review. Our methodology combines automated template-based QA generation with multistage linguistic re-engineering and iterative botanist review to ensure clinical accuracy and domain relevance. A comprehensive summary of the overall methodology is shown in Fig 1. We further benchmark the dataset by evaluating nine open-source vision-language models in a zero-shot setting, and demonstrate the dataset’s utility as a training resource through parameter-efficient fine-tuning of a compact multimodal model. To our knowledge PlantExpertVQA is the first multimodal dataset created under extensive guidance by domain experts for the sole purpose of model training and evaluation.

Overall, our primary contributions include:

1. **Large-scale, expert-verified VQA dataset grounded in a structured Disease Knowledge Base**: We introduce PlantExpertVQA, a multi-source domain-specific VQA dataset consisting of 765,186 expert-verified QA pairs grounded over 150,841 images, covering 38 crop species and 89 disease conditions, sourced from 45 open-access repositories under verified permissive licenses. The dataset is built on a structured Disease Knowledge Base, in which every crop-condition pair is encoded as a machine-readable card capturing pathogen taxonomy, transmission pathways, environmental risk factors, severity rubrics, organ-specific visual symptoms, visually similar lookalike conditions with key differentiating features, and management strategies. The dataset spans nine question categories organized into three levels of cognitive complexity, ranging from basic identification to causal and counterfactual reasoning.
2. **Two-Stage QA Generation Pipeline with Domain Expert Review**: We implement an automated template-based QA synthesis pipeline followed by a linguistically guided re-engineering phase to ensure semantic variety and answer diversity. The entire dataset underwent two phases of domain expert validation by experienced botanists, ensuring clinical accuracy and relevancy to the field.
3. **Benchmarking and Domain Adaptation**: We benchmark nine open-source vision-language models on PlantExpertVQA in a zero-shot setting and find that current frontier models perform poorly on the dataset. We further demonstrate, through parameter-efficient fine-tuning of a 2B-parameter model on a small fraction of the dataset, that PlantExpertVQA enables substantial improvements across all question categories, establishing its value as both a benchmark and a training resource.

These contributions make PlantExpertVQA a useful and reliable dataset for research in plant disease diagnosis using visual question answering.

## Methodology

### Multi-source image collection and preprocessing

To build a comprehensive and robust foundation for PlantExpertVQA, we curated a diverse collection of 45 open-source plant disease datasets. The compiled corpus extends substantially beyond the widely used PlantVillage repository, which alone contributed approximately 55,000 laboratory-controlled images of 14 crops; the remaining 44 datasets contribute field-acquired imagery across additional crop species and disease conditions, expanding the final corpus to 38 unique crop species and 89 disease conditions. A critical inclusion criterion for these datasets was the presence of an explicit, well-curated hierarchical directory structure similar to the meticulous organization found in the widely used PlantVillage repository. In these chosen datasets, each directory systematically encodes the image’s crop species and health status (e.g., Tomato__Late_blight or Apple__Healthy). This structural consistency was vital for ensuring accurate, automated data generation in subsequent stages. All source datasets were released under permissive open licenses (Creative Commons or equivalent), and the full provenance of every image including its originating dataset, license, and citation is preserved in the released metadata; a complete listing of the 45 source datasets is provided in Supplementary Table S1.

We implemented a systematic preprocessing pipeline across this multi-source compilation to guarantee standardization. We first eliminated exact and near-duplicate images using a combination of MD5 and Perceptual Hashing (pHash), applied both within and across source datasets to remove redundancy that arises when overlapping crop-disease pairs are independently distributed by multiple repositories. All images were then standardized to a uniform resolution. To prevent geometric distortion of crucial biological features, such as lesion shapes and leaf margins, we applied aspect-ratio-preserving padding rather than simple cropping or stretching, filling any remaining area with black pixels. Finally, we conducted automated technical audits to filter out low-quality samples and artifact biases. This included evaluating Laplacian variance to remove excessive blurriness, analyzing intensity histograms to discard over- or under-exposed images, and performing a background bias assessment using HSV color thresholding. This rigorous curation process resulted in a highly standardized, expert-chosen visual foundation of 150,841 high-quality images ready for QA generation.

### Disease knowledge base construction

**Fig. 2:**
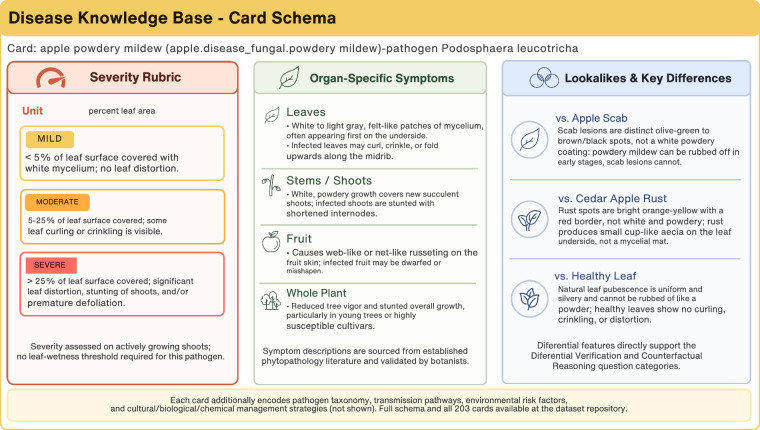
Structure of a Disease Knowledge Base card. Each of the 203 machine-readable cards encodes a specific crop–condition pair, detailing pathogens, symptoms, risk factors, and management. Illustrated here for apple powdery mildew are three key components: a severity rubric (left), organ-specific symptoms (centre), and lookalike conditions (right). This standardized schema directly grounds the generation of Visual Attribute, Severity, Differential, and Counterfactual questions.

To ground question and answer generation in established phytopathological science rather than in the priors of a general-purpose language model, we constructed a structured Disease Knowledge Base (KB) that encodes domain expertise in standardized json-schema. The KB consists of 203 cards, each corresponding to a single crop-condition pair drawn from the 38 crop species and 89 disease conditions present in the curated image collection; pairs include both healthy controls and pathological conditions of fungal, bacterial, viral, and abiotic etiology. The schema, illustrated in Fig. 2, was designed in collaboration with practising botanists and plant pathologists, and the substantive content of every card was extracted from established phytopathology literature^23,24^ and validated through expert consensus before inclusion.

Each card encodes seven structurally distinct components. *Pathogen taxonomy* records the kingdom-to-species classification of the causal agent, supporting the higher-order recall required by Specific Disease Identification questions. *Transmission pathways* document vector species, dispersal mechanisms, and overwintering reservoirs, and *environmental risk factors* record the temperature, humidity, and leaf-wetness conditions under which infection is favoured; together these components ground the answers to Causal Reasoning questions. The *severity rubric* defines mild, moderate, and severe grades against quantitative thresholds (e.g., percent leaf area affected) and provides the discriminative basis for Severity Assessment questions. *Organ-specific symptoms* catalogue canonical visual indicators separately for leaves, stems, fruit, roots, and the whole plant, providing the source descriptions for Visual Attribute Grounding questions. *Lookalike conditions* record visually similar diseases together with explicit *key differentiating features*, supporting Differential Verification and the negative cases of Counterfactual Reasoning. Finally, *management strategies* catalogue cultural, biological, and chemical interventions, supporting Comprehensive Description and management-related question types. Figure 2 illustrates three of these components the severity rubric, organ-specific symptoms, and lookalikes with key differences for the apple powdery mildew card.

We deliberately encoded this domain knowledge in a structured format rather than as free-form text. The schema enforces uniformity across all 203 cards, and this uniformity carries over to the questions and answers generated from them. It also makes every answer traceable to a specific KB field, which supports both auditability and reproducibility. Finally, it lets the same card be queried along several cognitive axes (visual attributes, causal mechanisms, severity, and differential diagnosis) without authoring each axis separately. The full schema and the complete set of 203 cards are released alongside the dataset.

### Programmatic QA generation from the knowledge base

Equipped with the structured Disease Knowledge Base described in the previous subsection, we generated the foundational pool of question–answer pairs by programmatically aligning each KB card with the images of the corresponding crop–condition pair. The hierarchical directory structure of the compiled image corpus, in which each image path explicitly encodes its crop and disease label, supplied the index linking each image to its KB card. For every (image, KB card) pair, our generation pipeline instantiated a fixed set of question templates, populating template slots with content drawn directly from the relevant KB fields: pathogen taxonomy, organ-specific symptoms, severity descriptors, lookalike comparisons, or management strategies, depending on the question category.

The question taxonomy comprises nine categories grouped into three levels of cognitive complexity. The categories range from foundational perception tasks such as Plant Species Identification, through detailed verification tasks such as Visual Attribute Grounding, to higher-order tasks such as Causal and Counterfactual Reasoning. The taxonomy was designed in consultation with three experienced graduate students from the Department of Botany, University of Dhaka, and was refined iteratively against early generation outputs. We deliberately avoided free-form generation by large language models at this stage: every question and every answer is traceable to an explicit KB field via a deterministic template, which eliminates hallucination risk and makes the entire foundational pool auditable and reproducible. The three levels and their constituent categories are shown in Supplementary Tables S2–S4 respectively (see Supplementary Information).

### Data refinement and re-engineering

Following the completion of programmatic generation, the foundational pool of 965,382 question–answer pairs was passed through a refinement pipeline whose purpose was twofold: to enrich the linguistic diversity of templated text without altering its meaning, and to remove non-verifiable or low-quality QA pairs through a combination of expert review and automated detection. Each stage of this pipeline is described in the subsections that follow.

#### Linguistic diversification through template-based paraphrasing

In order to introduce a more diverse vocabulary, we applied Template-Based Paraphrasing. We first searched for the top repetitive questions and answers appearing more than 10,000 times. We rephrased the same text multiple times while preserving its original meaning. For questions, we first identified the most frequent templates. Next we used a pool of 10–15 high-quality paraphrasers to manually edit these templates. These annotators operated under strict guidelines to diversify the syntactic structure and phrasing without altering canonical pathological terminology, crop identifiers, or severity descriptors. This constraint was essential to prevent semantic drift, ensuring that while the linguistic variety of the questions increased, their underlying pathological accuracy remained deterministically anchored to the original Knowledge Base. By locking these core entities, we captured natural conversational diversity without compromising scientific precision. The resulting paraphrase pools thus expanded surface-level variety while keeping every question semantically faithful to its source template.

An example question and its paraphrase pool are shown in Table 1. At this stage, our team of specialists reviewed question variations from all nine categories to ensure scientific accuracy and consistency. We discarded all grammatically incorrect and excessively complex questions. Once validated, we replaced each question template with a randomly selected variation from its paraphrase pool. This process expanded our dataset vocabulary by 83.1%, making the dataset more communicative and accessible.

**Table 1:** Question and its Paraphrase Pool.

Original Question	Paraphrase Pool
What disease does this [Crop] leaf have?	Identify the disease affecting this [Crop] leaf?
	Can you diagnose the ailment present on this [Crop] foliage?
	What pathological condition is evident on this [Crop] leaf?
	Which disease is indicated by the symptoms on this [Crop] leaf?
	Please specify the disease observed on this [Crop] leaf.

**Fig. 3:**
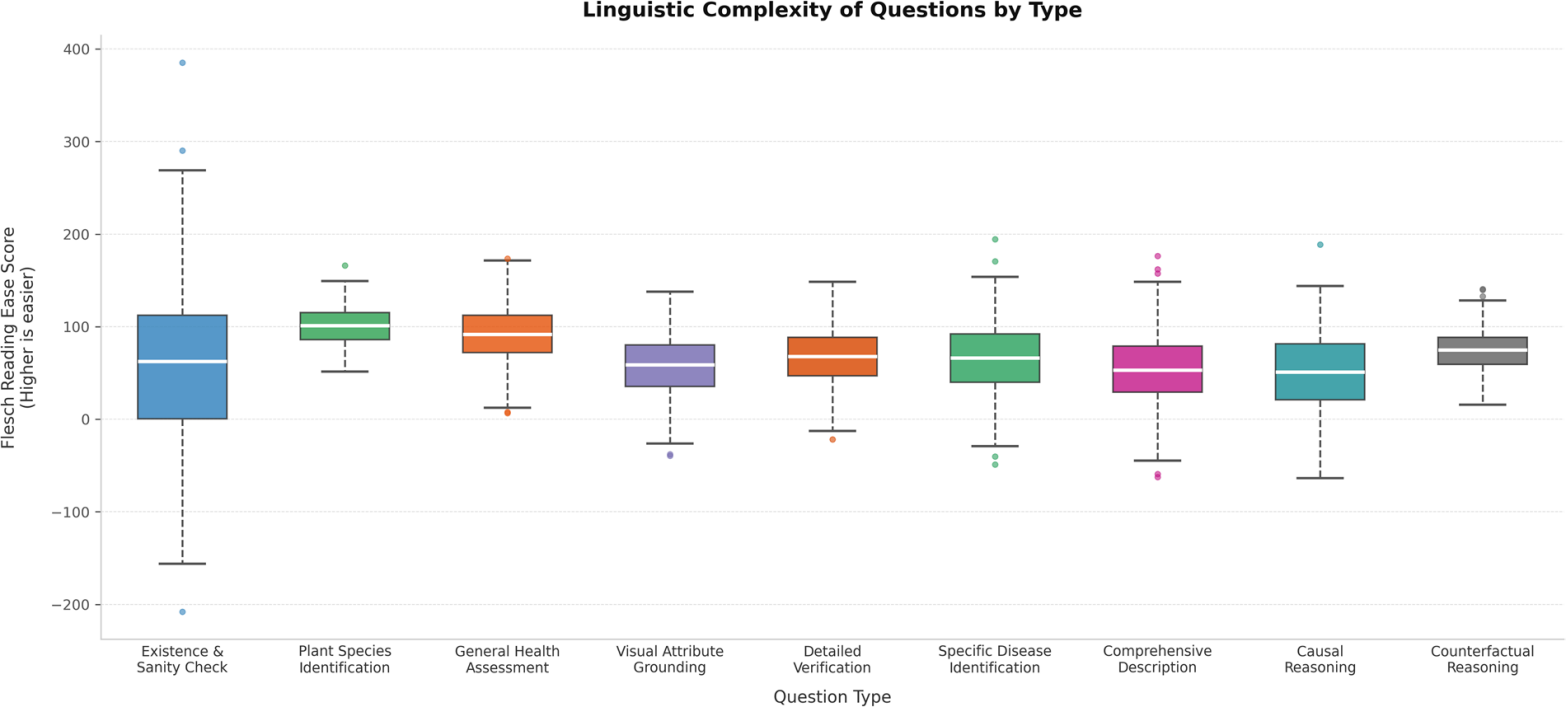
Question Linguistic Complexity.

We measured the comprehensibility of each question type through the Flesch Reading Ease Score. This score is calculated by considering average sentence length and the average number of syllables per word. Higher scores indicate better readability. Figure 3 shows that this score varies across all question categories, indicating that the dataset covers both simple and complex inquiries.

For answers, we employed a similar strategy. The dataset initially showed answer-side bias, in which a small fixed group of answers was provided for a given disease. To counter this, we first identified the “answer bundles” for each major disease. We then replaced them with a diverse pool of descriptive answers. For instance, all answers related to Late_blight (e.g., **“This is a leaf with Late_blight.”, “The cause is Late_blight.”**) were replaced by a random selection of more elaborative answers such as: **“The large, dark, water-soaked lesions are a key sign of Late Blight.”** and **“This is a classic presentation of Late Blight, caused by Phytophthora infestans.”**.

**Fig. 4:**
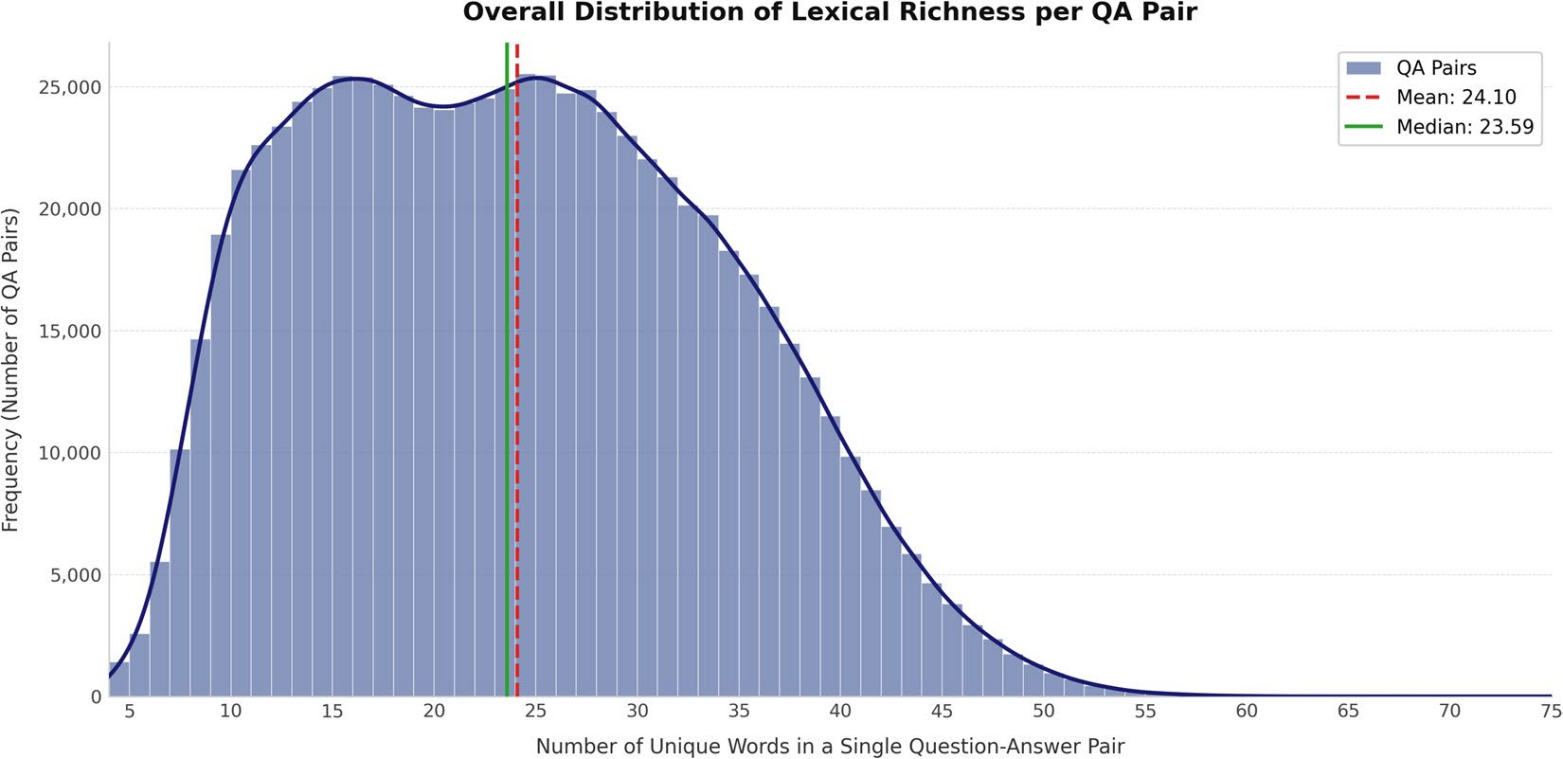
Lexical richness in QA pairs through re-engineering.

The number of unique words per QA pair was calculated through the Lexical Richness Score. Figure 4 shows a mean count of 24.1 unique words per QA pair, meaning each pair uses approximately 24 words. This indicates that the generated data has a rich range of vocabulary while maintaining clarity.

As a result the number of unique words in questions and answers increased as shown in Table 2. An image template with the associated paraphrased QA pairs across multiple question categories is shown in Fig. 7.

**Table 2:** Vocabulary Growth in Question and Answer Sets.

Metric	Initial	Final	Increase
Question Vocabulary	1,789	3,275	1,486
Answer Vocabulary	148	4,245	4,097

#### Targeted stratified undersampling for structural balance

Next we investigated if each question category was structurally balanced. We discovered that primarily four types of questions: **Visual Attribute Grounding,** **Detailed Verification,** **General Health Assessment** and **Plant Species Identification** contributed to the overall imbalance.

**Fig. 5:**
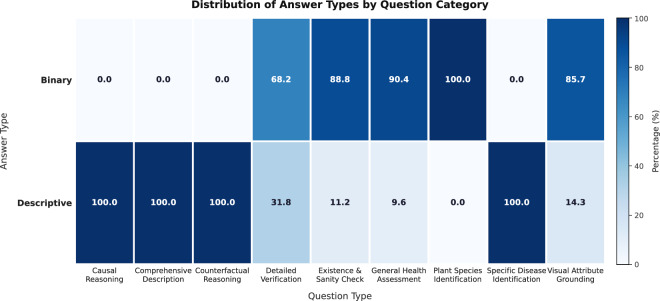
Heatmap of Answer type by Question Type.

Figure 5 shows the heatmap of the binary and descriptive answers per question category. It can be seen that all four aforementioned categories have above 80% binary answers. Further analysis showed that on average 62.6% of these questions were skewed toward negative answers. To solve this issue, we employed targeted stratified undersampling. Instead of undersampling the entire dataset, we first extracted the QA pairs only from the problematic question types. There, we retained 100% of the “Yes” answer pairs and randomly reduced the “No” answer pairs to create a balanced 40/60 binary ratio. The 40/60 ratio was a heuristic choice, employed specifically to preserve most of the dataset while also reducing the structural imbalance. Finally we merged the reduced QA bundles back into the original dataset. This process removed **139,905** QA pairs from the structurally imbalanced categories, resulting in an improved and balanced structure: the overall binary answer ratio being 59.4%. Although described here alongside the other refinement operations, this stratified balancing was applied as the final step of corpus construction, after the two expert-review phases; consequently the intermediate corpus of 905,182 QA pairs reported in the Technical Validation precedes balancing, and the 139,905 pairs removed at this stage are reflected in the final corpus total of 765,186 QA pairs.

## Data Records

The complete PlantExpertVQA dataset is publicly available on the Hugging Face Hub at 10.57967/hf/9145^25^ under a CC BY 4.0 licence. The deposit is organised into three components, the image corpus, the question–answer records, and the structured Disease Knowledge Base, together with the predefined train, validation and test partition described below.

**Images**. The 150,841 preprocessed images are provided in JPEG format. They are organised in a hierarchical directory structure in which each parent folder encodes the crop species and the health or disease status of its contents (e.g., Tomato__Late_blight, Apple__healthy), mirroring the convention of the source repositories. Every image carries a unique image_id that links it to the corresponding question–answer records and to its provenance metadata.

**Question–answer records**. The 765,186 QA pairs are released as tabular records (CSV, with an equivalent JSON Lines representation). Each record contains the following fields: qa_id (a unique identifier for the QA pair); image_id (the identifier of the grounding image); crop and disease (the crop species and condition); severity (the severity grade, where applicable); question_category (one of the nine taxonomy categories); cognitive_level (Level 1–3); question_text and answer_text (the natural-language QA pair); and split (train, validation, or test).

**Disease Knowledge Base**. The 203 crop–condition cards are provided as machine-readable JSON files, one per crop–condition pair. Each card exposes structurally distinct fields for pathogen taxonomy, transmission pathways, environmental risk factors, the severity rubric, organ-specific symptoms (catalogued separately for leaves, stems, fruit, roots, and the whole plant), lookalike conditions with their key differentiating features, and management strategies. Every answer in the QA records is traceable to a specific KB field, as detailed in the Methods.

**Data splits and provenance**. The corpus is partitioned at the image level into train (535,881 QA pairs; 70%), validation (76,384; 10%), and test (152,921; 20%) subsets; the partition is image-disjoint and is recorded both in the split field and as separate split files. The full provenance of every image and its originating source dataset, licence, and bibliographic citation is preserved as per-image metadata; the 45 constituent source datasets are enumerated in Supplementary Table S1.

## Technical Validation

To ensure dataset quality, we combined automated quality evaluation with two phases of expert review by experienced botanists. Given the extensive scale of the generated corpus, relying solely on manual inspection was logistically improbable, while purely automated metrics often fail to capture nuanced clinical inaccuracies. Therefore, we established a synergistic hybrid workflow: initial broad-scale human oversight identifies systemic patterns, which subsequently informs algorithmic outlier detection to pinpoint anomalies for a final, targeted manual review. The following sections describe each step of the validation pipeline.

### Domain expert review: Phase one

**Fig. 6:**
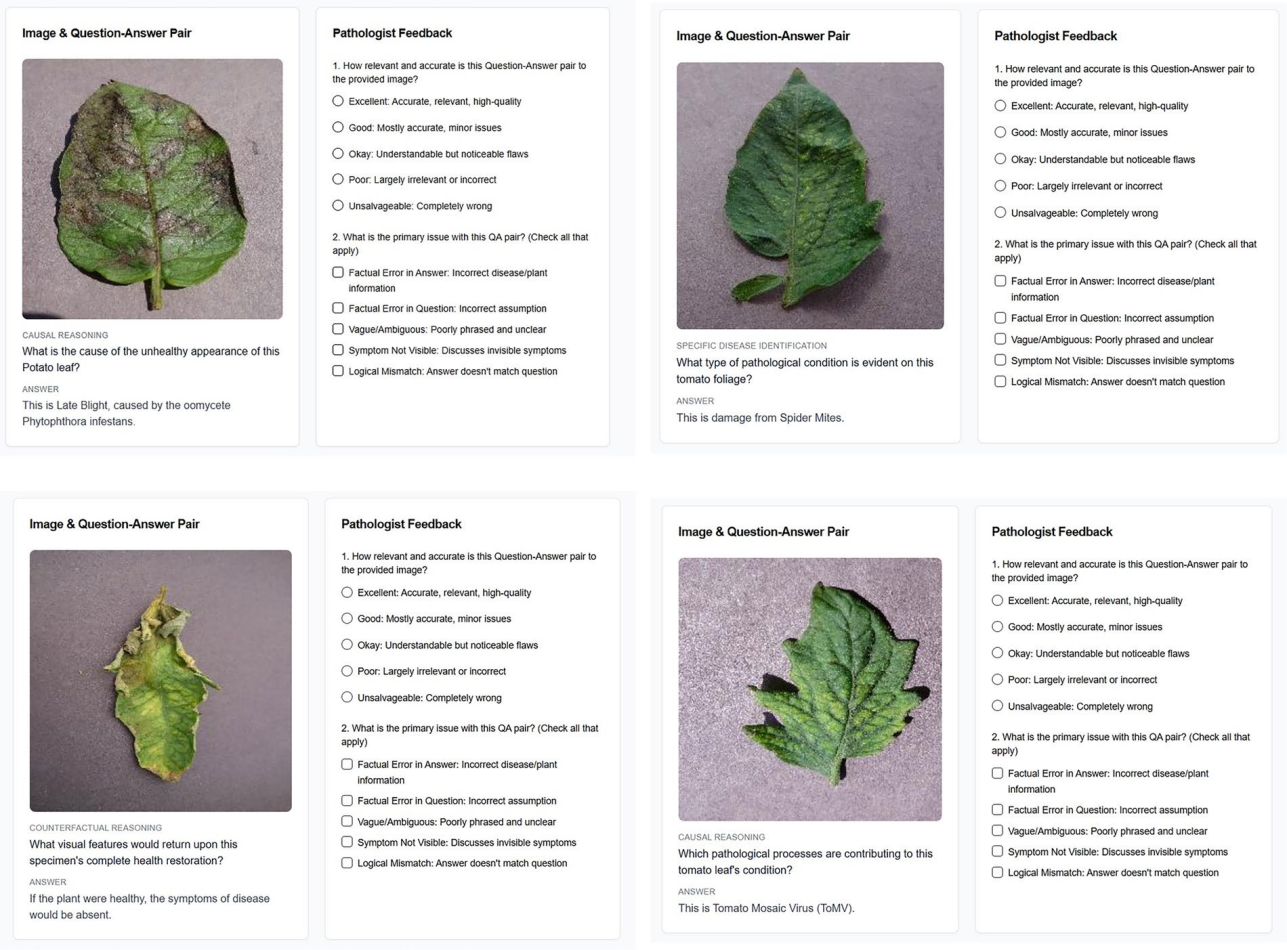
Phase One Expert Review Form.

The primary objective of this initial evaluation phase was to establish a baseline of scientific validity across the foundational question-answer pairs and intercept any recurring structural flaws. To facilitate this massive undertaking, we created a custom web interface hosting our entire dataset. This interface allowed a team of experienced botanists to efficiently review question-answer relevancy and identify related issues through a structured submission form. An example page from the website is shown in Fig. 6.

**Fig. 7:**
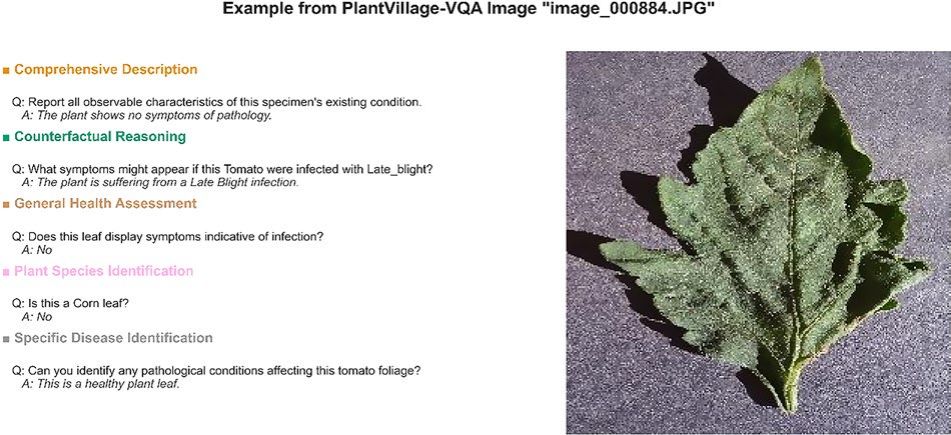
Image and its associated Questions.

Initial specialist feedback showed satisfactory performance across eight question categories. However, some associated answers relied heavily on generic fallback templates that contained no correlation with the questions. This was prevalent in the Counterfactual Reasoning category. While the questions themselves were correctly posed hypothetical scenarios (e.g., “What visual features would be different if this plant were healthy?”), their answers were occasionally paired with simple diagnostic statements (e.g., A: “The diagnosis is Tomato Yellow Leaf Curl Virus.”). We identified this problem as poor specificity in counterfactual answer generation. To address it, we applied a Hierarchical Correction Pipeline to the 94,500 counterfactual QA pairs whose answers relied on generic fallback templates.

#### Implementation of the hierarchical correction pipeline

First, we leveraged the dataset’s own internal knowledge to mine all 57,200 questions from the Visual Attribute Grounding category. These contained expert-phrased canonical descriptions of visual symptoms (e.g., “Does the leaf exhibit dark, concentric ‘bullseye’ rings?”). We programmatically extracted these descriptions and mapped them to key symptom words (e.g., ‘bullseye’ → “dark, concentric ‘bullseye’ rings”), creating a canonical_phrase_map.

To enhance counterfactual specificity we adopted a simple, defensible rule: fix when verifiable; delete when not. A QA pair was considered verifiable when it contained all three of the following: (i) valid crop_disease provenance, (ii) a disease_keyword that mapped to a condition, and (iii) an expert symptom phrase for that condition. If any element above was missing (e.g., a generic answer such as “The plant is healthy.”), the QA pair was deleted. We then regenerated the valid counterfactual answers using the template: “A healthy leaf would not show … [canonical symptom] …”. We refrained from using LLMs for question refinement to eliminate hallucination risk and preserve traceability. The numerical effect of the process on the counterfactual question category is shown in Table 3.

**Table 3:** Quantitative Outcome of Hierarchical Correction.

Category	Count
Initial Pool	94,500 QA Pairs
Corrected (Regenerated Pool)	34,300 QA Pairs (36.3%)
Deleted (Non-Verifiable)	60,200 QA Pairs (63.7%)

This ensured every surviving counterfactual answer was logically responsive, symptom-grounded, and reproducible from code and maps. A further 27,481 counterfactual pairs whose answers were deterministically grounded in the Disease Knowledge Base at generation time were exempt from this pipeline and merged with the 34,300 corrected pairs, yielding the 61,781 counterfactual pairs in the final corpus.

#### Impact of logical correction

The Hierarchical Correction reduced the model’s reliance on generic fallback answers, indicating a significant improvement in the counterfactual question category. Table 4 provides a quantitative comparison of the dataset before and after final refinement.

**Table 4:** Comparison of quality metrics before and after specialist feedback and correction.

Metric	Before Feedback	After Feedback	Improvement
Generic answers (% of counterfactuals)	55.03%	5.49%	↓ 90% reduction
Single generic template frequency	10,800	1,370	↓ 87% reduction

### Automated quality evaluation and outlier detection

After initial botanist review and correction, we conducted a thorough analysis of the entire database. We developed an automated pipeline to highlight suspicious QA pairs for expert re-evaluation. This process quantified abstract notions of quality, such as “simplicity”, “vagueness” and “relevance” using established techniques from natural language processing and information theory.

**Fig. 8:**
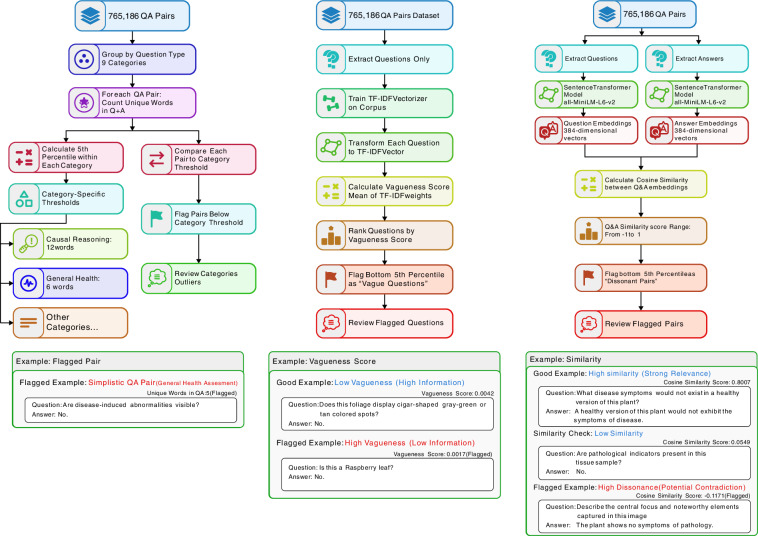
Automated quality evaluation and outlier detection workflow: relative simplicity (left), vagueness score assessment (center), and semantic dissonance (right).

Three distinct analyses were performed on the post-correction corpus of 905,182 QA pairs as shown in Fig. 8. These metrics do not indicate inaccuracy in questions, but low conversational ability. For example, the question “Is this a Raspberry leaf?” is short but not vague in intent. These analyses allowed us to conduct a rigorous recheck of the entire database for communicative value.

**Relative Simplicity:** We assessed relative simplicity by counting unique words per QA pair and comparing them with the mean unique-word counts of their question category. Questions below the 5th percentile threshold for each category (e.g., a Causal Reasoning question with 5 unique words) were flagged as outliers. The flowchart on the left of Fig. 8 shows this assessment.

**Vagueness Score:** We hypothesised that vague or low-value questions rely heavily on common words (e.g., “what”, “is”, “leaf”) and lack specific keywords. To quantify this, we calculated a vagueness score using the TF-IDF weight of every word in a question. Lower scores indicated less informative content, and the bottom 5th percentile of such questions were flagged. The center flowchart in Fig. 8 shows the process.

**Semantic Similarity:** To detect semantic alignment between QA pairs, we used sentence-transformer embeddings (all-MiniLM-L6-v2) and computed QA similarity via cosine similarity. Lower scores identified weaker question-answer relevancy. This process is shown in the right flowchart of Fig. 8. In total, 118,600 QA pairs were flagged by the outlier detection pipeline.

### Domain expert review: Phase two

**Fig. 9:**
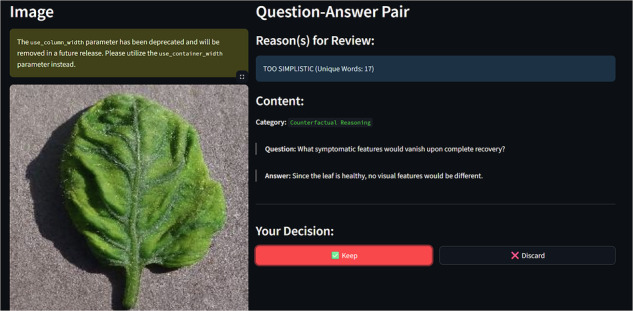
Phase Two Expert Review Form.

In phase two, we focused only on the 118,600 QA pairs flagged by the automated outlier detection pipeline. We created a second web interface that displayed samples from this compilation. This form allowed reviewers to either keep or discard each QA pair. An example page of the form is shown in Fig. 9.

During this final inspection, our team of botanists reviewed a random sample of 2,837 flagged forms, of which only 91 were discarded. This corresponds to a sample retention rate of 96.8%, indicating that the automated pipeline’s flagging was conservative and that the surviving corpus was ready for model application and benchmarking. These two phases of expert validation, supported by the automated outlier detection pipeline, yielded the final PlantExpertVQA corpus of 765,186 high-quality QA pairs.

## Comparison with Existing Plant-Disease VQA Datasets

To position PlantExpertVQA within the existing landscape of agricultural visual question answering resources, we compare it against the principal plant-disease VQA datasets reported in the recent literature. The comparator set comprises PlantVillageVQA, the v1 release that PlantExpertVQA extends; CDDM^26^, the largest published plant-disease VQA dataset prior to this work; and CDwPK-VQA^27^, a knowledge-guided dataset constructed under a methodologically similar premise to ours but at smaller scale. Table 5 summarises the comparison along seven dimensions covering scale, coverage, source diversity, generation methodology, and expert validation.

**Table 5:** Comparison of PlantExpertVQA against existing plant-disease VQA datasets and benchmarks.

Dataset	QA Pairs	Images	Crops / Cond	Answer Format	Reasoning Depth	KB Grounding	Expert Validation
AgroBench^43^	NR	NR	203 / 682	Constrained (MCQ)	Multi-task (7 topics)	×	Expert Agronomist
LeafNet^44^	13,950	186,000	22 / 62	Constrained (MCQ)	Visual Identification	×	Author-reported
AgMMU^45^	1,492 / 5,460^†^	12,481	Broad Ag.	MCQ & Brief Open	Factual Dialogic	Partial (AgBase)	USDA-Verified
CDDM^26^	~1,000,000	137,000	16 / 60	Short Open (GPT-4 gen.)	Surface Visual	×	Author-reported
CDwPK-VQA^27^	22,320	2,748	10 / 19	Brief Open	Multi-attribute	Partial (prior knowledge)	Author-reported
WheatRust-VQA^46^	NR	1,800^‡^	1 / 4	Fixed Templated	Symptom Identification	×	Author-reported
**PlantExpertVQA (Ours)**	**765,186**	**150,841**	**38 / 89**	Open-ended (Multi-sentence)	Causal, Counterfactual, Severity	Extensive (203-Card KB)	Two-phase Botanist

Consequently, PlantExpertVQA uniquely bridges the gap between the sheer scale of LLM-generated datasets and the reliability of knowledge-grounded resources. We note that recent vision-language systems like AgroGPT^21^ and LLaVA-PlantDiag^22^ are excluded from this direct comparison; as instruction-tuned models trained on LLM-generated data without disclosed expert validation protocols, they are not standalone benchmark datasets.

## The Final PlantExpertVQA Corpus

Before proceeding to model evaluation, we summarise the composition of the released PlantExpertVQA corpus across four orthogonal axes: scale, coverage, question-category distribution, and the released splits. Table 6 consolidates these statistics. With 765,186 expert-verified question–answer pairs grounded over 150,841 images, PlantExpertVQA averages 5.07 QA pairs per image, a ratio that supports multi-faceted evaluation of a single visual instance across cognitive levels rather than the one-question-per-image regime that characterises classification-style VQA datasets such as CDDM^26^. Coverage extends across 38 crop species and 89 disease conditions, yielding 203 unique crop × condition combinations, the largest such cardinality reported for an expert-validated plant-disease VQA dataset to our knowledge.

**Table 6:** Composition of the final PlantExpertVQA corpus.

**Scale**	
Total QA Pairs	765,186
Total Images	150,841
Average QA Pairs per Image	5.07
**Coverage**	
Unique Crop Species	38
Unique Disease Conditions	89
Unique Crop × Condition Combinations	203
**Question Categories**	
Specific Disease Identification	135,009 (17.64%)
Comprehensive Description	116,424 (15.22%)
Plant Species Identification	108,447 (14.17%)
Causal Reasoning	89,986 (11.76%)
Detailed Verification	78,829 (10.30%)
General Health Assessment	66,572 (8.70%)
Counterfactual Reasoning	61,781 (8.07%)
Existence & Sanity Check	60,457 (7.90%)
Visual Attribute Grounding	47,681 (6.23%)
**Splits** (image-level stratified)	
Train	535,881 (70%)
Validation	76,384 (10%)
Test	152,921 (20%)

Question-category counts follow the nine-category taxonomy of Supplementary Tables S2–S4, aggregated post-refinement, and sum to the headline corpus total. Train, validation, and test splits are image-level stratified and image-disjoint, preserving crop and disease distributions across splits.

The distribution of QA pairs across the nine-category taxonomy reflects the cognitive structure introduced in the Methodology. Higher-order reasoning categories occupy a substantial share of the corpus: Specific Disease Identification (17.64%), Comprehensive Description (15.22%), and Causal Reasoning (11.76%) together account for over 44% of all QA pairs, ensuring that the corpus is weighted toward the open-ended generative tasks that current vision-language models find most challenging^28,29^. Conversely, foundational perception categories such as Existence & Sanity Check (7.90%) and Visual Attribute Grounding (6.23%) remain represented in sufficient volume to support discriminative pretraining and reliability auditing, but do not dominate the distribution as is common in classification-style benchmarks. This deliberate weighting follows the design principle articulated in Bloom-style hierarchical evaluation frameworks^30^, in which higher-order cognitive tasks warrant proportionally greater representation when the explicit aim is to evaluate reasoning rather than perception alone.

The corpus is released with a 70/10/20 train, validation, and test partition stratified at the image level. Stratification is performed across question category, source dataset, crop species, and disease condition simultaneously, and the resulting splits are image-disjoint such that no image appears in more than one partition. This choice is deliberate: question-level stratification, which permits different QA pairs derived from the same image to fall into different splits, has been shown to overstate generalisation in multi-question-per-image VQA settings by leaking visual content across the train–test boundary. The image-level partition adopted here eliminates that risk and provides a conservative testbed against which subsequent vision-language models can be benchmarked and fine-tuned, as we demonstrate in the following section.

## Evaluation and Benchmark

### Evaluation metrics

We evaluate model outputs using six complementary metrics that together capture lexical overlap, sequence-level alignment, and semantic similarity between generated answers and ground-truth references.

**Exact Match (EM)** measures the fraction of predictions that match the reference answer exactly after case normalisation:

1 $${\rm{EM}}=\frac{1}{N}\mathop{\sum }\limits_{i=1}^{N}{\bf{1}}[{\hat{y}}_{i}={y}_{i}]$$

where ${\hat{y}}_{i}$ is the model prediction, *y*_*i*_ is the reference, and *N* is the number of QA pairs. EM rewards precise short-form answers and is most informative for closed-set questions such as crop or disease identification.

**Token-F1** computes the harmonic mean of token-level precision and recall between the prediction and reference token sets, following the SQuAD evaluation convention^31^:

2 $${\rm{F}}1=\frac{2\cdot |\hat{T}\cap T|}{|\hat{T}|+|T|}$$

where $\hat{T}$ and *T* denote the multisets of tokens in the prediction and reference. Token-F1 relaxes the strict equality requirement of EM while still penalising both omissions and spurious tokens.

**BLEU-***n*^32^ measures *n*-gram precision between the prediction and reference, with a brevity penalty to discourage degenerate short outputs:

3 $$\begin{array}{ll}\mathrm{BLEU}-n=\mathrm{BP}\cdot \exp (\mathop{\sum }\limits_{k=1}^{n}{w}_{k}\,\log \,{p}_{k}), & \mathrm{BP}=\min \,(1,{e}^{1-r/c})\end{array}$$

where *p*_*k*_ is modified *n*-gram precision of order *k*, *w*_*k*_ are uniform weights, *c* is the prediction length, and *r* is the reference length. We report BLEU-1 and BLEU-2 to capture vocabulary and short-phrase overlap.

**ROUGE-L**^33^ is computed as the F1-measure over the longest common subsequence (LCS) between prediction and reference:

4 $$\begin{array}{lll}{\rm{ROUGE}}-{\rm{L}}=\frac{(1+{\beta }^{2})\cdot {R}_{{\rm{LCS}}}\cdot {P}_{{\rm{LCS}}}}{{R}_{{\rm{LCS}}}+{\beta }^{2}\cdot {P}_{{\rm{LCS}}}}, & {R}_{{\rm{LCS}}}=\frac{|{\rm{LCS}}(\hat{y},y)|}{|y|}, & {P}_{{\rm{LCS}}}=\frac{|{\rm{LCS}}(\hat{y},y)|}{|\hat{y}|}\end{array}$$

ROUGE-L captures sequence-level alignment without requiring contiguous matches, which makes it well suited to long-form, free-word-order pathology descriptions.

**BERTScore**^34^ computes semantic similarity through cosine alignment of contextual token embeddings produced by a pretrained language model. Given embeddings ${{\bf{x}}}_{i}$ for the reference and ${\hat{{\bf{x}}}}_{j}$ for the prediction:

5 $$\begin{array}{ll}{P}_{\mathrm{BERT}}=\frac{1}{|\hat{y}|}\sum _{{\hat{x}}_{j}\in \hat{y}}\mathop{\max }\limits_{{x}_{i}\in y}\,{{\bf{x}}}_{i}^{{\rm{{\rm T}}}}{\hat{{\bf{x}}}}_{j}, & {R}_{\mathrm{BERT}}=\frac{1}{|y|}\sum _{{x}_{i}\in y}\mathop{\max }\limits_{{\hat{x}}_{j}\in \hat{y}}\,{{\bf{x}}}_{i}^{{\rm{{\rm T}}}}{\hat{{\bf{x}}}}_{j}\end{array}$$

with *F*_BERT_ defined as their harmonic mean. We adopt this metric to capture semantic equivalence between paraphrased outputs and references which is a property that purely lexical metrics cannot measure.

These metrics were selected to span the full evaluation spectrum: from strict surface-form agreement (EM, BLEU) through sequence-level structural alignment (ROUGE-L, Token-F1) to semantic equivalence (BERTScore). Together they support a comprehensive characterisation of model performance across the heterogeneous answer-length distributions present in PlantExpertVQA, ranging from single-word disease names to multi-sentence diagnostic descriptions. All metrics were computed on the complete test set, ensuring full coverage of the question-category, crop, and disease-condition distributions present in PlantExpertVQA.

### Zero-shot benchmarking of vision-language models

We evaluated PlantExpertVQA against nine vision-language models spanning four principal architectural lineages: contrastive dual-encoders (CLIP^35^), classification-style VQA models (BLIP^36^), Q-Former-bridged hybrids (BLIP-2^37^, InstructBLIP^28^), and instruction-tuned multimodal LLMs (LLaVA-1.5/1.6^29,38^, Qwen-VL series^39,40^, Gemma-3^41^).

Despite their pretraining scale, these frontier models struggle on PlantExpertVQA (Table 7). The strongest model, Gemma-3-4B-IT, attains only 15.26 ROUGE-L and 11.20 BERTScore-F1 against expert reference descriptions. Performance broadly scales with architectural modernity: dual-encoders and classification models cluster near 7 ROUGE-L, Q-Former hybrids and early MLLMs around 9–10, and recent instruction-tuned models reach 13–15. Consequently, a substantial gap to reference-quality pathology generation remains across all lineages.

**Table 7:** Zero-shot evaluation of nine vision–language models on PlantExpertVQA.

Model	Evaluation Metrics (%)						Avg Len
	EM	Token-F1	BLEU-1	BLEU-2	ROUGE-L	BERTScore	
Gemma-3-4B-IT	2.04	17.72	31.19	4.77	**15.26**	**11.20**	11.6
Qwen3-VL-2B-Instruct	3.29	**17.88**	**37.18**	**6.63**	14.54	8.53	17.0
Qwen2-VL-2B-Instruct	**8.91**	13.76	36.57	3.23	13.16	7.95	3.2
LLaVA-1.6 Mistral-7B	0.90	14.07	24.42	4.36	10.26	7.26	27.5
LLaVA-1.5-7B	7.39	10.21	26.87	0.58	9.87	3.15	2.5
InstructBLIP Vicuna-7B	6.54	9.70	28.53	2.18	9.40	−0.92	2.5
BLIP-2 FlanT5-XL	3.57	9.92	28.20	2.13	8.98	−2.74	5.7
CLIP ViT-L/14	3.31	7.44	15.30	2.27	6.95	3.48	7.1
BLIP VQA Large	5.43	6.65	21.41	0.04	6.64	−1.59	1.3

All metric values are percentages; Avg Len denotes average prediction length in tokens.

Two key observations characterize this difficulty. First, Exact Match (EM) and ROUGE-L produce inverted rankings: Qwen2-VL leads in EM (8.91) by producing precise short answers, while Gemma-3 leads in ROUGE-L via longer descriptions, indicating no single architecture seamlessly handles both closed-set and long-form reasoning. Second, high BLEU-1 scores often pair with low or negative BERTScores (e.g., BLIP-2), demonstrating that superficial lexical overlap does not equate to semantic accuracy. Ultimately, mastering PlantExpertVQA requires domain-grounded, semantically precise generation which is a capability current open-source models lack without explicit domain adaptation.

### Domain adaptation through parameter-efficient fine-tuning

To determine whether the zero-shot performance gap stems from fundamental architectural limits or missing domain knowledge, we conducted parameter-efficient fine-tuning on Qwen3-VL-2B-Instruct^40^. This model was selected for three reasons. First, it was the strongest 2B-class zero-shot baseline (14.54 ROUGE-L). This provides a robust starting point to unambiguously measure adaptation gains. Second, its architecture couples a language backbone to a Vision Transformer via a lightweight projector. This design represents the dominant paradigm in modern instruction-tuned models, which ensures our findings generalize. Third, its 2B parameter scale aligns with the computational and memory constraints of real-world agricultural decision-support systems. Such systems require edge-deployable efficiency.

Adaptation was performed using Low-Rank Adaptation (LoRA)^42^, with rank-64 adapters applied to the query, key, value, and feedforward projection matrices of the language backbone. The vision encoder remained frozen, yielding 69.7 million trainable parameters –3.17% of the model’s 2.20 billion total. Training was conducted on a 500-image subset (2,337 QA pairs) of the PlantExpertVQA training split, stratified across question category, source, crop, and disease condition to mirror the evaluation distribution and verified to be image-disjoint from the test set. The deliberately small training set was designed to test whether the structured disease knowledge encoded in PlantExpertVQA provides sufficient supervision under limited data, rather than to maximise absolute performance. Optimisation used AdamW with a learning rate of 2 × 10^−4^ and cosine decay over three epochs; the best checkpoint by validation loss was retained for evaluation.

Table 8 contrasts the fine-tuned model against the strongest zero-shot performer on PlantExpertVQA, Gemma-3-4B-IT, which led the zero-shot benchmark on both ROUGE-L and BERTScore-F1 despite operating at twice the parameter count. The fine-tuned 2B model exceeds Gemma-3-4B-IT by 4.0× on ROUGE-L (61.70 vs. 15.26), 5.7× on BERTScore-F1 (63.27 vs. 11.20), and 9.2× on exact match (18.85 vs. 2.04), with comparable margins across all remaining metrics. Average prediction length converges to 27.2 tokens, closely matching the reference distribution and indicating that the model has internalised the long-form pathology description style of PlantExpertVQA rather than continuing to default to short factual outputs.

**Table 8:** Performance of fine-tuned Qwen3-VL-2B against the strongest zero-shot performer on PlantExpertVQA.

Setting	Evaluation Metrics (%)						Avg Len
	EM	Token-F1	BLEU-1	BLEU-2	ROUGE-L	BERTScore	
zero-shot (Best)	2.04	17.72	31.19	4.77	15.26	11.20	11.6
Qwen3-VL-2B-FT	**18.85**	**64.65**	**66.18**	**41.74**	**61.70**	**63.27**	27.2

All metric values are percentages; Avg Len denotes average prediction length in tokens. **Bold** marks the best result on each metric.

Decomposed by question category in Table 9, the improvement is most pronounced on precisely the question types that resisted zero-shot adaptation across all evaluated systems: General Health Assessment rises from a best zero-shot value of 6.72 to 90.00 ROUGE-L, Visual Attribute Grounding from 27.06 to 86.30, Causal Reasoning from 21.64 to 74.48, and Comprehensive Description from 10.43 to 63.57. Two conclusions follow. First, the diagnostic-reasoning failures observed in zero-shot evaluation reflect a knowledge gap rather than a capability gap; once the structured pathological content of PlantExpertVQA is exposed to the model, it is acquired efficiently and produces marked downstream improvements. Second, PlantExpertVQA functions effectively both as a benchmark and as a training resource, parameter-efficient adaptation on a fraction of one percent of the training split suffices to elevate a compact 2B-parameter model above the strongest zero-shot models in our benchmark, including those at substantially larger parameter counts. We frame these results as a proof of concept for the utility of PlantExpertVQA in domain adaptation; comprehensive fine-tuning across additional architectures and at the full training scale is left to future work.

**Table 9:** Per-category ROUGE-L for fine-tuned Qwen3-VL-2B against the strongest zero-shot performer per category.

Question Category	Best Zero-shot	Leading Model	Fine-tuned Qwen3-VL-2B	Gain
Plant Species Identification	39.68	Qwen3-VL	**57.56**	+17.88
General Health Assessment	6.72	BLIP-2	**90.00**	+83.28
Visual Attribute Grounding	27.06	Qwen3-VL	**86.30**	+59.24
Detailed Verification	24.32	Qwen3-VL	**62.67**	+38.35
Specific Disease Identification	7.10	CLIP	**57.96**	+50.86
Comprehensive Description	10.43	Qwen3-VL	**63.57**	+53.14
Causal Reasoning	21.64	Qwen3-VL	**74.48**	+52.84
Counterfactual Reasoning	21.69	Qwen3-VL	**62.95**	+41.26

Results are reported across eight of the nine question categories defined in the PlantExpertVQA taxonomy; the Existence & Sanity Check category is omitted as it serves a binary image-validity filter and is not separately evaluated during fine-tuning. The best zero-shot ROUGE-L across all nine evaluated models is reported for each category, with the leading model named in the adjacent column. **Bold** marks fine-tuned results; Gain = Fine-tuned − Best zero-shot. Values for Specific Disease Identification, Comprehensive Description, Causal Reasoning, and Counterfactual Reasoning are unweighted means over their constituent question sub-types.

### Comparison with existing plant-disease VQA models

Existing plant-disease vision-language models report performance under widely heterogeneous evaluation protocols, which complicates direct head-to-head comparison. Table 10 summarises the reported performance of the principal comparator systems against our fine-tuned model.

**Table 10:** Comparison of reported performance across plant-disease VQA models.

System	Test Set	Task Formulation	Reported Metric	Value
**PlantExpertVQA Qwen3-VL-2B-FT (ours)**	PlantExpertVQA	Open-ended generation, 9 categories	ROUGE-L	**61.70**
			BERTScore-F1	**63.27**
			Token-F1	**64.65**
			Exact Match	**18.85**
AgroGPT^21^	AgroEvals (6 tasks)	Multi-task agricultural QA	Task-level accuracy	Multi-task
LLaVA-PlantDiag^22^	PlantVillage-derived labels	Disease classification	Classification accuracy	~96.0
CDDM Swin-T5^26^	CDDM (292 unique answers)	Closed-set VQA	Classification accuracy	~99.94
CDwPK-VQA^27^	CDwPK-VQA (22,320 pairs)	Closed-set VQA	Accuracy	86.06

Each system is evaluated on its own test distribution under its own metric, illustrating the methodological fragmentation in the field. Reported values for comparator systems are taken from the respective publications.

Two observations follow from Table 10. First, all comparator systems report classification-style accuracy against discrete answer sets, whereas PlantExpertVQA evaluates open-ended generation against multi-sentence pathology references; the two regimes measure substantively different capabilities and should not be ranked on a single axis. Second, the apparent absolute performance of comparator systems is partly an artefact of constrained answer spaces, most visibly in the case of CDDM, whose test set contains only 292 unique answers across approximately 1 million QA pairs. PlantExpertVQA addresses this fragmentation by providing a public unified benchmark spanning nine question categories with open-ended ground-truth references, supporting future evaluations in which competing systems can be retrained or zero-shot evaluated under identical conditions.

## Limitations

Several limitations of the present work warrant explicit acknowledgement. First, the fine-tuning experiment is intentionally scoped as a proof of concept: a single model architecture (Qwen3-VL-2B) was adapted on a small subset (2,337 QA pairs) rather than at full training scale, and comprehensive comparison across architectures and at the full training scale remains future work. Second, the dataset is monolingual, with all questions and answers in English, which limits direct applicability in agricultural contexts where local languages dominate; multilingual extension is a natural next step. Third, the source-dataset compilation, although diverse across 45 repositories, inherits the geographic and crop-distribution biases of its constituent datasets, with disease conditions affecting temperate and subtropical crops better represented than those of tropical and arid regions. Fourth, the Disease Knowledge Base, although extensive, is not exhaustive: 89 disease conditions cover the most prevalent pathologies in the source imagery but exclude rare, emerging, or geographically restricted conditions, and the static schema does not yet reflect ongoing taxonomic revisions or newly characterised pathogens. Finally, model performance is evaluated through automated lexical and semantic metrics; a complementary human evaluation by practising plant pathologists would provide further evidence of clinical utility and is left to future work.

## Generative AI Usage

The authors used a generative AI tool to help edit the language of this manuscript and improve its readability. It was not used to produce any of the data, results, or scientific claims. All text was checked by the authors, who take full responsibility for the final content.

## Supplementary information

Supplementary Information

## Data Availability

The PlantExpertVQA dataset, comprising all 150,841 preprocessed images, the 765,186 expert-verified question–answer pairs, and the 203-card Disease Knowledge Base, is publicly available on the Hugging Face Hub at https://huggingface.co/datasets/SyedNazmusSakib/PlantExpertVQA^25^ under a CC BY 4.0 licence. A detailed description of the constituent files, their formats, and their fields is provided in the Data Records section.

## Code availability

The code for the programmatic QA generation pipeline, the data-refinement and template-paraphrasing steps, the automated outlier-detection pipeline, and the parameter-efficient fine-tuning experiments reported in this work is publicly available at https://github.com/syed-nazmus-sakib/PlantExpertVQA.
