# Supplementary material for: A Visual Question Answering Dataset for Benchmarking Vision-Language Models in Plant Science
Source: Sci Data. 2026 Aug 10;13:1161. doi: 10.1038/s41597-026-07779-y (PMC13458762; doi:10.1038/s41597-026-07779-y)
Supplement: Supplementary file 1 [file 41597_2026_7779_MOESM1_ESM.pdf]

Supplementary Information

S1. Constituent Open-Source Plant Disease Datasets

The visual foundation of PlantExpertVQA is a compilation of 45 publicly released plant disease image datasets, enumerated in Table S1. The compilation was assembled to extend substantially beyond the widely cited PlantVillage corpus [1, 46], which despite its foundational role in the literature is restricted to 14 crop species captured under laboratory-controlled conditions. The remaining 44 datasets were drawn predominantly from recent Mendeley Data releases and contribute field-acquired imagery across additional crops, geographic origins, and pathological conditions, addressing the well-documented domain gap between controlled and in-field plant pathology imagery noted in prior work [47]. Each constituent dataset was admitted to the compilation on the basis of two criteria: (i) the presence of an explicit hierarchical directory structure in which the parent folder encodes both the crop species and the health or disease status of every image, enabling automated label-preserving integration with the structured Disease Knowledge Base described in the main text; and (ii) release under a permissive open licence (CC0 or CC BY 4.0) compatible with redistribution and derivative scientific use. The full provenance of every image in the released corpus, including its originating dataset, licence terms, and bibliographic citation, is preserved in the released metadata.

Table S1: Constituent open-source plant disease image datasets aggregated to form the visual foundation of PlantExpertVQA. Each entry reports the dataset name as released by its authors, the primary crop or crop group covered, the licence under which the dataset was released, and the corresponding bibliographic reference.

#	Dataset Name	Primary Crop(s)	Licence	Ref.
1	PlantVillage	Multi-crop (14)	CC0	[1]
2	Plant Pathology Challenge 2020	Apple	CC BY 4.0	[2]
3	Apple Leaf Diseases (ICAR-CITH)	Apple	CC BY 4.0	[3]
4	Chili Plant Leaf Disease and Growth Stage	Chili	CC BY 4.0	[4]
5	Banana and Banana Leaf Dataset	Banana	CC BY 4.0	[5]
6	CAIR-BGD-2025: Bottle Gourd Disease and Growth Stages	Bottle gourd	CC BY 4.0	[6]
7	Plant Leaf Disease Recognition Dataset	Multi-crop	CC BY 4.0	[7]
8	Papaya Leaf Disease Image Dataset	Papaya	CC BY 4.0	[8]
9	Real-World Papaya Leaf (Bangladeshi Orchards)	Papaya	CC BY 4.0	[9]
10	Eggplant Leaf Disease Dataset	Eggplant	CC BY 4.0	[10]
11	Eggplant Comprehensive Dataset	Eggplant	CC BY 4.0	[11]
12	High-Resolution Eggplant Leaf Dataset	Eggplant	CC BY 4.0	[12]
13	BrinjalFruitX	Brinjal (eggplant)	CC BY 4.0	[13]
14	High-Resolution Lychee Plant Diseases	Lychee	CC BY 4.0	[14]
15	BDLitchi	Litchi	CC BY 4.0	[15]
16	LitchiLeaf4001	Litchi	CC BY 4.0	[16]
17	TLD-BD: Tea Leaf Dataset	Tea	CC BY 4.0	[17]
18	Advanced Tea Crop Disease Study	Tea	CC BY 4.0	[18]
19	Hibiscus and Tea Leaf Dataset	Hibiscus, Tea	CC BY 4.0	[19]

Continued on next page

Table S1 continued from previous page

#	Dataset Name	Primary Crop(s)	Licence	Ref.
20	Hibiscus Leaf Diseases Classification	Hibiscus	CC BY 4.0	[20]
21	Multi-crop Leaf Disease (Bitter gourd, Okra, Pumpkin, Ridge gourd)	Multi-crop	CC BY 4.0	[21]
22	SAR-CLD-2024: Cotton Leaf Disease	Cotton	CC BY 4.0	[22]
23	Cotton Leaf Image Dataset for Disease Classification	Cotton	CC BY 4.0	[23]
24	MoringaLeafNet	Moringa	CC BY 4.0	[24]
25	MangoLeafBD	Mango	CC BY 4.0	[25]
26	Comprehensive Mango Leaf Disease Dataset	Mango	CC BY 4.0	[26]
27	Sunflower Fruits and Leaves	Sunflower	CC BY 4.0	[27]
28	Sunflower Plant Health and Growth Stage	Sunflower	CC BY 4.0	[28]
29	Multifaceted Rose Leaf Disease	Rose	CC BY 4.0	[29]
30	Tomato Disease Dataset	Tomato	CC BY 4.0	[30]
31	Tomato Leaf Diseases	Tomato	CC BY 4.0	[31]
32	Tomato Leaf (Multiclass Detection and Classification)	Tomato	CC BY 4.0	[32]
33	Tomato (Bangladesh High-Resolution)	Tomato	CC BY 4.0	[33]
34	AJLCD-2025: Arabian Jasmine Leaf Condition	Arabian jasmine	CC BY 4.0	[34]
35	Durian Leaf Diseases (Vietnam)	Durian	CC BY 4.0	[35]
36	BDRubberLeaf	Rubber	CC BY 4.0	[36]
37	Cauliflower Diseases Identification	Cauliflower	CC BY 4.0	[37]
38	CitrusLeafVision (Lemon)	Lemon	CC BY 4.0	[38]
39	Jackfruit Plant Leaf Disease	Jackfruit	CC BY 4.0	[39]
40	AgriLeafNet (Fruit Tree Leaves)	Multi-fruit	CC BY 4.0	[40]
41	Pisum sativum (Pea) Image Dataset	Pea	CC BY 4.0	[41]
42	Turmeric Plant Leaf Disease	Turmeric	CC BY 4.0	[42]
43	NGLD: Niphad Grape Leaf Disease	Grape	CC BY 4.0	[43]
44	Burmese Grape Leaf Disease	Burmese grape	CC BY 4.0	[44]
45	UGV: Guava Leaves Disease Bangladesh	Guava	CC BY 4.0	[45]

In aggregate, the 45 constituent datasets contribute approximately 321,000 raw images, of which 150,841 images survived the standardisation, de-duplication, and quality-audit pipeline described in the main text (Methodology, *Multi-Source Image Collection and Preprocessing*). After consolidation against the Disease Knowledge Base, the surviving corpus spans 38 unique crop species and 89 distinct disease conditions, with overlapping crop–condition pairs across multiple source datasets resolved through perceptual-hash-based cross-dataset de-duplication. The dominant geographic origin of the compilation is South Asia, with a substantial concentration of datasets curated in Bangladesh, India, and Vietnam; this regional emphasis is acknowledged as a limitation in the main text and represents a target for future expansion towards under-represented tropical and arid agro-ecological zones. We note that the heavy reliance on recent Mendeley Data releases reflects a broader trend in agricultural computer vision towards open-access, field-acquired imagery [47], and we anticipate that the compilation strategy adopted here will remain extensible as further such datasets become available.

S2. Detailed Question Category Taxonomy

This section provides the detailed specification of the nine-category question taxonomy referenced in the Methodology. The taxonomy is organised into three levels of cognitive complexity, following the design principle articulated in Bloom-style hierarchical evaluation frameworks for multimodal comprehension [50]: foundational perception and identification (Level 1), detailed analysis and verification (Level 2), and higher-order reasoning and inference (Level 3). For each category we report (i) its diagnostic purpose, (ii) the Disease Knowledge Base (KB) field or fields from which template slots are instantiated, and (iii) two to three representative templates drawn from the released generation script. Templates are shown in their canonical pre-paraphrase form; the linguistically diversified surface forms produced by the re-engineering pipeline are released alongside the dataset.

S2.1 Level 1: Foundational Perception and Identification

Level 1 questions assess the model's capacity to identify discrete elements in the image with factual, closed-set answers. They establish baseline visual competence and act as sanity filters: a model that fails Level 1 questions cannot reasonably be expected to succeed at higher cognitive levels. The three categories are summarised in Table S2.

Question Category	Purpose and KB Grounding	Representative Templates
**1. Existence & Sanity Check**	This category filters out irrelevant or background images and verifies that the model recognises plant material as the primary subject. Templates are content-independent and do not require KB instantiation.	*What is the primary subject of this image?*<br>*Does this image depict plant material?*<br>*Is this a photograph of a plant or a plant part?*
**2. Plant Species Identification**	For species-specific diseases, identifying the host plant is a prerequisite for downstream diagnosis. This category tests fine-grained visual categorisation. Templates are instantiated using the `crop.common_name` field of the corresponding KB card, with both positive and negative variants generated to produce a balanced binary task.	*Is this a [Crop] leaf?*<br>*Which crop species is depicted in this image?*<br>*Can you identify the host plant shown here?*
**3. General Health Assessment**	This category tests the model's ability to make a high-level binary judgement of the plant's overall health status. Templates are instantiated from the `issue_type` field of the KB card, which distinguishes diseased from healthy entries.	*Is the plant in this image healthy?*<br>*Does this leaf show any signs of disease or stress?*<br>*Would you classify the depicted specimen as healthy or diseased?*

Table S2: Question categories for Level 1 (Foundational Perception and Identification). KB fields refer to the schema introduced in the Methodology.

S2.2 Level 2: Detailed Analysis and Verification

Level 2 questions move from coarse identification to symptom-level analysis. They constitute the diagnostic core of PlantExpertVQA: the model must detect, and ground its responses in, the specific visual symptoms catalogued in the phytopathology literature [48, 49]. The two categories are summarised in Table S3.

Question Category	Purpose and KB Grounding	Representative Templates
**1. Visual Attribute Grounding**	This category tests whether the model can detect specific, often subtle, visual symptoms that are referenced in a textual phrase. Templates are instantiated from the `symptoms` fields of the KB card, which catalogue canonical visual indicators separately for leaves, stems, fruit, roots, and the whole plant. The underlying symptom descriptions are sourced from established phytopathology literature [48, 49] and validated through expert consensus during KB construction.	*Does the leaf exhibit dark, concentric "bullseye" rings?*<br>*Are yellow halos visible around the lesions on this leaf?*<br>*Is there a powdery white coating on the leaf surface?*
**2. Detailed Verification**	This category combines species identification and disease identification into a single compositional binary query. Templates jointly populate `crop.common_name` and `condition.common_name` from the KB card. Both positive (correct crop and disease) and negative (mismatched crop, mismatched disease, or both) variants are generated.	*Is this [Crop] leaf infected with [Disease_Name]?*<br>*Does this image show a [Crop] plant affected by [Disease_Name]?*<br>*Can [Disease_Name] be diagnosed from the symptoms visible on this [Crop] leaf?*

Table S3: Question categories for Level 2 (Detailed Analysis and Verification). KB fields refer to the schema introduced in the Methodology.

S2.3 Level 3: Higher-Order Reasoning and Inference

Level 3 questions require the model to synthesise information across multiple visual cues, infer relationships between symptoms and underlying mechanisms, and reason about hypothetical or counterfactual scenarios. These are the open-ended generative tasks on which current vision-language models exhibit the largest performance gaps in our zero-shot evaluation. The four categories are summarised in Table S4.

Question Category	Purpose and KB Grounding	Representative Templates
**1. Specific Disease Identification**	This is the direct open-ended diagnostic task. Unlike Detailed Verification, the model is not given a candidate disease; it must recall and provide the correct one from its internal knowledge. Answers are sourced from the `condition.common_name` field of the KB card.	*Please provide a diagnosis for the condition shown.*<br>*What specific disease is affecting this plant?*<br>*Identify the pathological condition visible on this leaf.*
**2. Comprehensive Description**	This category tests the model's ability to generate holistic summary descriptions that span multiple aspects of the image. Answers are composed by aggregating multiple KB fields (pathogen, key organ-specific symptoms, and severity grade) into a single descriptive paragraph.	*Provide a full description of the plant and its condition.*<br>*Describe the plant, the visible symptoms, and the likely diagnosis.*<br>*Summarise the visual evidence and provide a clinical assessment.*
**3. Causal Reasoning**	This category probes whether the model can infer the causal agent and the conditions responsible for the visible symptoms. Answers are instantiated from the `condition.pathogen`, `transmission`, and `environmental_risk_factors` fields of the KB card.	*What is the cause of the unhealthy appearance of this leaf?*<br>*Which pathogen is responsible for the symptoms shown?*<br>*Under what environmental conditions does this disease typically develop?*
**4. Counterfactual Reasoning**	This category tests the model's capacity to reason about a hypothetical state that is contrary to the visual evidence. Answers are constructed by negating the symptom descriptions of the affected KB card, e.g., "a healthy leaf would not show ... [canonical symptom]... ", ensuring counterfactual answers are grounded in the same KB field that drives Visual Attribute Grounding. This shared grounding underpins the Hierarchical Correction Pipeline described in the main text.	*If this plant were healthy, what visual features would be different?*<br>*Which of the visible features would be absent in a healthy specimen?*<br>*What changes would indicate that this plant has recovered from infection?*

Table S4: Question categories for Level 3 (Higher-Order Reasoning and Inference). KB fields refer to the schema introduced in the Methodology.

Across the three levels, every template in the released generation pipeline is mapped deterministically to a specific KB field or combination of fields. This design choice eliminates the hallucination risk associated with free-form generation by large language models [51] and renders the entire foundational pool of 965,382 QA pairs auditable and reproducible from the released KB and template scripts. The post-refinement category counts and their proportions within the final 765,186-pair corpus are reported in the corpus-composition table of the main manuscript (section "The Final PlantExpertVQA Corpus").

References

[1] David P. Hughes and Marcel Salathé. An open access repository of images on plant health to enable the development of mobile disease diagnostics through machine learning and crowdsourcing. *arXiv preprint arXiv:1511.08060*, 2015.

[2] Ranjita Thapa, Kai Zhang, Noah Snavely, Serge Belongie, and Awais Khan. The Plant Pathology Challenge 2020 data set to classify foliar disease of apples. *Applications in Plant Sciences*, 8(9):e11390, 2020.

[3] ICAR-CITH. Apple Leaf Diseases Image Dataset of ICAR-CITH. *Mendeley Data*, 2024. doi:10.17632/gm6mfz8fz6.1.

[4] Md Asraful Sharker Nirob, A K M Fazlul Kobir Siam, Prayma Bishshash, and Md Assaduzzaman. Chili Plant Leaf Disease and Growth Stage Dataset from Bangladesh. *Mendeley Data*, V1, 2025. doi:10.17632/w9mr3vf56s.1.

[5] Utsab Das, Showrov Azam, and Md Abdullah Al Kafi. Banana and Banana Leaf Dataset for Classification and Disease Detection. *Mendeley Data*, V2, 2025. doi:10.17632/5nfjzntwd8.2.

[6] Md Asraful Sharker Nirob, Prayma Bishshash, A K M Fazlul Kobir Siam, and Mayen Uddin Mojumdar. CAIR-BGD-2025: Annotated Dataset for Bottle Gourd Disease and Growth Stages. *Mendeley Data*, V1, 2025. doi:10.17632/x6rpc5nzzm.1.

[7] Tanvir Ahmed, Mst Taposi Rabeya Taposi, Marzanul Alam Mukhor, and Mayen Uddin Mojumdar. Plant Leaf Disease Recognition Dataset. *Mendeley Data*, V1, 2024. doi:10.17632/5g238dv4ht.1.

[8] Hasan Al Banna, Md. Fuad Hossain, and Mayen Uddin Mojumdar. Papaya Leaf Disease Image Dataset. *Mendeley Data*, V1, 2024. doi:10.17632/3kwgxg4stb.1.

[9] Mohammad Rifat Ahmmad Rashid, Raiyan Gani, Jubaer Ahmed, Maherun Nessa Isty, and Sawkat Ali. Healthy and Unhealthy Papaya Leaf Images from Bangladeshi Orchards. *Mendeley Data*, V1, 2024. doi:10.17632/44p8v6ywsm.1.

[10] Maruful Islam Rafe, Farhan Masud Nayem, Shanto Babu Sarker, and Abdullah Al Shiam. Eggplant Leaf Disease Dataset. *Mendeley Data*, V2, 2025. doi:10.17632/mn8vfr9bw2.2.

[11] Md Asraful Sharker Nirob, Prayma Bishshash, Mariyam Bin Ayan, Tania Khatun, and Mohammad Shorif Uddin. Eggplant Dataset: A Comprehensive Dataset for Agricultural Research and Disease Detection. *Mendeley Data*, V1, 2024. doi:10.17632/5drkk544k8.1.

[12] Rakib Hasan, Sanimun Hossain Sanzit, Md Mamun Hosen, Ferdous Hasan, Md Mehedi Hasan Topu, and Muksitul Islam. High-Resolution Eggplant Leaf Image Dataset for Plant Disease Classification and Detection. *Mendeley Data*, V6, 2025. doi:10.17632/ss63ftnjnh.6.

[13] Md. Zahid Hasan, Abu Kowshir Bitto, and Md Hasan Imam Bijoy. BrinjalFruitX: A Field-Collected Image Dataset for Machine Learning and Deep Learning-Based Disease Identification in Brinjal Fruits. *Mendeley Data*, V1, 2025. doi:10.17632/ngc58fsxgd.1.

[14] Shahariar Hossain, Saifuddin Sagor, and Faruk Ahmed. High-Resolution Images of Lychee Plant Diseases for Classification and Detection. *Mendeley Data*, V2, 2025. doi:10.17632/52sstfpf5p.2.

[15] Kouser Ahamed, Rokonozzaman Ayon, Mst. Momotaz Begum, and Israt Jahan. BDLitchi: A Field-Collected Bangladeshi Litchi Leaf Disease Dataset for Deep Learning-Based Detection and Classification. *Mendeley Data*, V1, 2025. doi:10.17632/jhb24mszdk.1.

[16] Md. Taufiq Hasan, Sabbir Hossain Durjoy, Md Emon Shikder, Md. Safaet Zahangir, Md Muntasir Uddin, Md Mehedi Hasan Shoib, and Md Hasan Imam Bijoy. LitchiLeaf4001: A Comprehensive Dataset of Lychee Leaf Diseases for AI-Based Visual Diagnosis. *Mendeley Data*, V1, 2025. doi:10.17632/c3n2tc6jf4.1.

[17] Faruk Ahmed and Ferdous Ahmed. TLD-BD: A Comprehensive Tea Leaf Image Dataset for Leaf Condition Analysis. *Mendeley Data*, V2, 2025. doi:10.17632/d2xybhfw59.2.

[18] Md Hasan Ahmad. Advanced Tea Crop Disease Study: High-Resolution Dataset for Precision Agriculture and Pathological Insight. *Mendeley Data*, V4, 2024. doi:10.17632/tt2smzrzrs.4.

[19] Md Masum Billah, Saifuddin Sagor, and Mohammad Shorif Uddin. A Real-World Hibiscus and Tea Leaf Image Dataset for Classification. *Mendeley Data*, V1, 2025. doi:10.17632/5bzy89brkv.1.

[20] Md Masum Billah, Saifuddin Sagor, and Mohammad Shorif Uddin. Hibiscus Leaf Diseases Classification Dataset. *Mendeley Data*, V2, 2025. doi:10.17632/k8pp8cbp8k.2.

[21] Md Forhadul Islam, Imon Sutradhar, and Md Mizanur Rahman. Leaf Image Dataset for Disease Detection in Bitter Gourd, Okra, Pumpkin, and Ridge Gourd. *Mendeley Data*, V2, 2025. doi:10.17632/2svdj3yyrk.2.

[22] Prayma Bishshash, Md Asraful Sharker Nirob, Md Habibur Shikder, and Afjal Sarower. SAR-CLD-2024: A Comprehensive Dataset for Cotton Leaf Disease Detection. *Mendeley Data*, V2, 2024. doi:10.17632/b3jy2p6k8w.2.

[23] Shamim Ripon, Raiyan Gani, Nazratan Mazumder Niha, Wasimul Bari Rahat, Shafaeat Hasan Toufiq, Mushfida Ferdous Maisha, and Jubaer Ahmed. Cotton Leaf Image Dataset for Disease Classification. *Mendeley Data*, V1, 2025. doi:10.17632/t9hgvk2h9p.1.

[24] Abid Khan, Sabit Ahamed Preanto, Tapon Paul, and Md Hasan Imam Bijoy. MoringaLeafNet: A Multi-Class Leaf Disease Dataset for Precision Agriculture and Deep Learning Research. *Mendeley Data*, V3, 2025. doi:10.17632/w8sr775pjb.3.

[25] S. Ali, M. Ibrahim, S. I. Ahmed, M. Nadim, M. Rahman, M. M. Shejunti, and T. Jabid. MangoLeafBD: A comprehensive image dataset to classify mango leaf diseases. *Mendeley Data*, 2022. doi:10.17632/hxsnvwty3r.1.

[26] Md Faysal Hossan and Md Zamirul Islam Reyad. Comprehensive Mango Leaf Images Dataset for Multi-Class Disease Classification and Automated Plant Disease Detection. *Mendeley Data*, V2, 2025. doi:10.17632/jjhykb7v9w.2.

[27] Aditya Rajbongshi, Umme Sara, Bonna Akter, Rashiduzzaman Shakil, and Sadia Sazzad. Sun Flower Fruits and Leaves Dataset for Sunflower Disease Classification through Machine Learning and Deep Learning. *Mendeley Data*, V1, 2022. doi:10.17632/b83hmrzth8.1.

[28] Saifuddin Sagor, Md Faysal Hossan, Faruk Ahmed, and Md Zamirul Islam Reyad. Sunflower Plant Health and Growth Stage Image Dataset for Agricultural Machine Learning Applications. *Mendeley Data*, V1, 2025. doi:10.17632/y3ygk98ngr.1.

[29] Md Hasan Ahmad. Multifaceted Rose Leaf Disease Dataset for AI-Driven Plant Pathology. *Mendeley Data*, V1, 2024. doi:10.17632/8jtfk9szbg.1.

[30] Yongbo Liu. Tomato Disease Dataset. *Mendeley Data*, V1, 2025. doi:10.17632/c2x8rynybg.1.

[31] Ismail Hossain, Khandaker Rezoanul Haque, Abdullah Al Rafi, and Md Zahidul Islam Talukder. Tomato Leaf Diseases. *Mendeley Data*, V1, 2025. doi:10.17632/93h9p62kg4.1.

[32] Ahmed Imtiaz, Fahad Bin Islam Swapnil, Syed Rayhan Masud, and Debajoyti Karmaker. Tomato Leaf Dataset: A Dataset for Multiclass Disease Detection and Classification. *Mendeley Data*, V1, 2024. doi:10.17632/bpfd9cns5g.1.

[33] Puja Bapari, Md Zinnahtur Rahman Zitu, and Mst Ummehani. A High-Resolution Image Dataset of Tomato (*Solanum lycopersicum*) Leaves for Multi-Class Disease Detection and Classification from Bangladesh. *Mendeley Data*, V1, 2025. doi:10.17632/74zvtzx9pr.1.

[34] Rokonozzaman Ayon, Nur Yea Afroz Suchi, Md Asif Shahriar Arpon, Tanjina Ahmed Tuly, and Abdullah Al Noman. Arabian Jasmine Leaf Condition Dataset (AJLCD-2025). *Mendeley Data*, V1, 2025. doi:10.17632/nwdzwk89zs.1.

[35] Nguyen Thanh Truong, Xuan Linh Nguyen, Pham Dinh Cap Thang, and Tuong Le. A Durian Leaf Image Dataset of Common Diseases in Vietnam for Agricultural Diagnosis. *Mendeley Data*, V4, 2025. doi:10.17632/pxzvksbwnj.4.

[36] Pulak Deb Nath, Faruk Ahmed, and Belal Uddin. BDRubberLeaf: A Comprehensive Dataset of Rubber Tree Leaf Diseases from Bangladesh for Agricultural Research. *Mendeley Data*, V4, 2025. doi:10.17632/4kjz78m7x5.4.

[37] Md Mafiul Hasan Matin, Mehedi Hasan Hasan, and Sabik Ur Rahman. Cauliflower Diseases Identification Image Dataset. *Mendeley Data*, V2, 2025. doi:10.17632/c2hrwntzgh.2.

[38] Pulak Deb Nath. CitrusLeafVision: A Diverse Dataset for Lemon Leaf Disease Detection. *Mendeley Data*, V1, 2025. doi:10.17632/4dmgghmj3n.1.

[39] Rezwan Huq, Farzia Hossain, Shahida Begum, Raiyan Gani, and Jubaer Ahmed. Image Datasets for Jackfruit Plant Leaf Disease. *Mendeley Data*, V2, 2025. doi:10.17632/cggx64sj7m.2.

[40] Md Ehsanul Haque and Md Al-Imran. AgriLeafNet: Fruit Tree Leaf Dataset for Agricultural Research. *Mendeley Data*, V1, 2025. doi:10.17632/fkkfx4vdm3.1.

[41] Sandip Thite and Kailas Patil. *Pisum sativum* Image Dataset: Healthy and Disease-Affected Cases. *Mendeley Data*, V1, 2025. doi:10.17632/nnv3k3m94k.1.

[42] Md Riyad Hossain, Mohammad Rifat Ahmmad Rashid, Tasfia Binte Jahangir, Md Samir Hossain, Md Mahamudur Rahman, Raiyan Gani, Jubaer Ahmed, Raihan Ul Islam, and M Saddam Hossain Khan. Image Dataset for Turmeric Plant Leaf Disease Detection. *Mendeley Data*, V2, 2025. doi:10.17632/jtttfbx342.2.

[43] Madhuri Dharrao, Deepak Dharrao, Rakesh Sonawane, and Nilima Zade. Niphad Grape Leaf Disease Dataset (NGLD). *Mendeley Data*, V5, 2025. doi:10.17632/8nnd2ypcv3.5.

[44] Salman Af Rahman, Md Nafiz Imtiaz, Naima Ahmed, and Md Hasan Imam Bijoy. Burmese Grape Leaf Disease Dataset for Computer Vision-Based Plant Health Diagnosis. *Mendeley Data*, V1, 2025. doi:10.17632/k6gy38xv89.1.

[45] Sadib Hassan, Md Zahid Akon, Partho Sarathi Sarker, Rahat Hossain, Jannatul Ferdous, Md Mahadi Hasan Shaon, and Md Abdur Razzak. UGV: Guava Leaves Disease Dataset Bangladesh. *Mendeley Data*, V3, 2025. doi:10.17632/cc3rttngdr.3.

[46] Sharada P. Mohanty, David P. Hughes, and Marcel Salathé. Using deep learning for image-based plant disease detection. *Frontiers in Plant Science*, 7:215232, 2016.

[47] Davinder Singh, Naman Jain, Pranjali Jain, Pratik Kayal, Sudhakar Kumawat, and Nipun Batra. PlantDoc: A dataset for visual plant disease detection. In *Proceedings of the 7th ACM IKDD CoDS and 25th COMAD*, pages 249–253, 2020.

[48] George N. Agrios. *Plant Pathology*. Elsevier, 2005.

[49] Richard N. Strange and Peter R. Scott. Plant disease: a threat to global food security. *Annual Review of Phytopathology*, 43(1):83–116, 2005.

[50] Yunhao Gong and others. BloomVQA: Assessing Hierarchical Multi-modal Comprehension. *arXiv preprint*, 2024.

[51] Xiang Liu, Zhaoxiang Liu, Huan Hu, Zezhou Chen, Kohou Wang, Kai Wang, and Shiguo Lian. A multimodal benchmark dataset and model for crop disease diagnosis. In *Computer Vision – ECCV 2024*, pages 157–170. Springer Nature Switzerland, 2024.
